# Death of Prof. Barbour

**Published:** 1849-07

**Authors:** 


### DEATH OF PROF. BARBOUR.

Among the victims to cholera, in St. Louis, we are pained to see announced the name of Dr. Barbour, Professor of Obstetrics, in the University of Missouri. Prof. Barbour was a son of the late Governor Barbour, of Virginia. He was, for some time, a practitioner of medicine in Tennessee, and was thence called to a chair in the University of Missouri. As a lecturer he was fluent, earnest, and instructive, and as a practitioner he enjoyed a large share of public confidence. He wrote and published several papers which indicated sound judgment and practical tact, and were well received by the profession. To his other excellences, Dr. Barbour added the virtues of the Christian. He was a leading and active member of the Presbyterian church. In all the walks of life in which he was known, his influence was exerted for good, and his loss will be felt by the church as well as the school of which he was a member. We saw Prof. B. here in the spring. He was returning from a visit to his native State, in good health, and full of hope. With bright prospects before him, in the discharge of sacred professional duties, he has been suddenly cut down.

